# Genomic analysis of *Listeria monocytogenes* strains from dairy products in Ethiopia

**DOI:** 10.3389/fbinf.2025.1572241

**Published:** 2025-04-16

**Authors:** Mebrie Zemene Kinde, Bizuayehu Kerisew, Tegegne Eshetu, Abebe Tesfaye Gessese

**Affiliations:** ^1^ Department of Veterinary Biomedical Sciences, College of Veterinary Medicine and Animal Sciences, University of Gondar, Gondar, Ethiopia; ^2^ Department of Biology, Bahir Dar University, Bahir Dar, Ethiopia; ^3^ Department of Biomedical and Laboratory Science, College of Medicine and Health Science, University of Gondar, Gondar, Ethiopia

**Keywords:** antibiotic resistance genes, dairy products, *Listeria monocytogenes*, mobile genetic elements, virulence genes

## Abstract

This study explored virulence genes, antibiotic resistance genes, and mobile genetic elements in 14 *Listeria monocytogenes* strains from milk and dairy products collected from different regions of Ethiopia. The strains were classified into two Multilocus Sequence Typing sequence types (ST2 and ST45) and further grouped into clonal complexes (CC2) and different cgMLST types. Twenty-nine virulence genes were identified across all 14 strains, with *lplA1* detected at higher levels in all strains except SAMN28661660. All *L. monocytogenes* strains also carried four antibiotic resistance genes (*fosX*, *lin*, *norB*, *mprF*), contributing to their ability to withstand multiple antimicrobial agents. Notably, no plasmids or mobile genetic elements were detected. Stress resistance genes, including *stress survival islet 1* (*SSI1_lmo0447*), *lmo 1800*, and *lmo1799*, were identified in all strains. However, genes encoding for disinfectant resistance were not identified from all strains. LGI-2 was found in all the strains and none of the studied strains harbored LGI-1 and LGI-3. Conserved CRISPR-Cas systems were found in some strains. KEGG pathway analysis revealed that *inlA* and *inlB* genes facilitate bacterial internalization through host actin polymerization. Overall, the study provided crucial insights into the genomic features of *L. monocytogenes* in the Ethiopian dairy chain. It is crucial to establish continuous monitoring of *L. monocytogenes* in dairy products, improve sanitation, enforce stricter antibiotic usage and food safety regulations, and raise public awareness of associated risks.

## 1 Introduction

*Listeria monocytogenes* (*L. monocytogenes*) is a Gram-positive, facultative anaerobic bacterium responsible for listeriosis, which carries significant public health and economic implications (Rogalla & Bomar, 2023; Matle et al., 2020). This bacterium affects both humans and animals, spreading primarily through contaminated food, particularly unpasteurized dairy (Maury et al., 2019; Ravindhiran et al., 2023). In animals, it can cause severe conditions like encephalitis, septicemia, and abortion, especially in cattle and sheep (Matle et al., 2020). Meanwhile, in humans, symptoms range from mild gastroenteritis to severe conditions such as septicemia and meningitis, with high fatality rates among vulnerable populations like pregnant women, neonates, the elderly, and immunocompromised individuals (Hansen et al., 2006; Rohilla et al., 2024; Ravindhiran et al., 2023).

The pathogenicity of *L. monocytogenes* is influenced by key genes, such as those encoding the *internalins* (*InlA*, *InlB*), *listeriolysin O* (*LLO*), and actin assembly-inducing protein (ActA). These genes facilitate host cell invasion, intracellular survival, and intercellular spread (Orsi et al., 2011). Notably, these virulence factors can be dispersed throughout the genome islands (Quereda et al., 2021). With advances in genotyping and whole-genome sequencing, researchers have revealed considerable variation in pathogenic potential among *L. monocytogenes* isolates (Kathariou et al., 2017). Additionally, adaptation to stress in food processing environments can further enhance *L. monocytogenes* pathogenicity, as stress exposure may induce lasting phenotypic changes (Quereda et al., 2021).

Although antimicrobial chemotherapy is the primary treatment for listeriosis (Luque-Sastre et al., 2018), the increasing emergence of multidrug-resistant *L. monocytogenes* presents a growing public health challenge, particularly in developing countries like Ethiopia (Dufailu et al., 2021). The frequent use of antibiotics in food animal production has contributed to this resistance, leading to transmission to humans through contaminated dairy and meat products (Odu and Okonko, 2017). Alarmingly, resistance to antibiotics commonly used to treat listeriosis, such as tetracycline, ampicillin, and gentamicin, has been reported (Luque-Sastre et al., 2018). This rise in resistance underscores a severe threat to routine medical procedures, with potential deaths escalating if effective antibiotics become unavailable (Naghavi et al., 2024).

In Ethiopia, phenotypic antimicrobial resistance studies have highlighted the emergence of resistant *L. monocytogenes*. For instance, isolates from raw cow milk exhibited 100% resistance to nalidixic acid and high resistance to erythromycin (88%) (Tola, 2024). Similarly, resistance rates in sheep meat were high chloramphenicol (88.9%). Furthermore, clinical isolates from pregnant women demonstrated significant resistance to penicillin G and clindamycin (66.7%) (Tola, 2024). These findings signal a major public health challenge in a resource-limited country like Ethiopia.

Compounding the issue, *L. monocytogenes* is particularly concerning due to its ability to form biofilms on surfaces such as stainless steel and plastic in food processing facilities. This ability protects the bacteria from environmental stress and cleaning agents (Quereda et al., 2021; Ravindhiran et al., 2023). Inadequate sanitation in Ethiopian dairy facilities and poor handling practices, like insufficient storage temperatures, further exacerbate food safety risks (Abdi Keba et al., 2020; Mebrate et al., 2020). The genetic adaptability of *L. monocytogenes*, including the acquisition and loss of genetic elements, influences traits such as antibiotic resistance and environmental fitness (Dobrindt et al., 2004; Ghaly and Gillings, 2022; Wein et al., 2021).

On a global scale, *L. monocytogenes* remains a significant foodborne pathogen. The WHO reports that 20%–30% of clinical listeria infections are fatal, with approximately 23,150 infections and 5,463 deaths worldwide (World Health Organization, 2015). Developed countries like the U.S. report around 1,600 cases annually, with a mortality rate of 15%–20% (Datta and Burall, 2018). In Africa, the prevalence of listeriosis is likely underestimated due to limited diagnostic capabilities and inadequate surveillance data from farm animals (Sibanda et al., 2023). For example, the 2017 South African outbreak linked to contaminated processed meats resulted in over 1,000 cases and more than 200 deaths, highlighting the urgent need for improved food safety measures across the continent (Thomas et al., 2020). In Ethiopia, contamination rates of *L. monocytogenes* in milk and dairy products have reached up to 60% in some studies (Tola, 2024; Seyoum et al., 2015). This indicates that widespread dairy consumption poses a significant public health risk.

Despite the clear public health risks associated with *L. monocytogenes*, genomic research on this pathogen in Ethiopia remains limited. Most studies have focused on isolation and phenotypic characterization and Multilocus Sequence Typing (MLST) (Wei et al., 2024), leaving a gap in understanding the genetic diversity, virulence factors, and resistance genes specific to the local context. Consequently, there is an urgent need for genomic analysis of *L. monocytogenes* isolates to better understand their virulence and antimicrobial resistance profiles. Insights gained from studying the pathogen’s virulence, resistance mechanisms, and adaptation to environmental stress could guide the development of effective, cost-efficient antimicrobial agents and biocontrol strategies. Hence, this study aimed to conduct a genomic analysis of *L. monocytogenes* isolates from milk and dairy products in Ethiopia, focusing on identification of virulence and antimicrobial resistance genes, and mobile genetic elements (MGEs, genetic materials that can move within or between genomes, including across different organisms) linked to virulence and resistance from whole genome sequence.

The study identified multiple virulence genes in the *L. monocytogenes* strains, including *inlA* and *inlB*, which are crucial for host cell invasion. Additionally, *LGI-2* was found in all the strains and none of the studied strains harbored *LGI-1* and *LGI-3*. The isolates were categorized into two sequence types (ST2 and ST45) and further grouped into clonal complexes (CC2). All strains harbored antibiotic resistance genes, such as *fosX*, *lin*, *norB*, and *mprF*. However, MGEs and genes related to metal resistance, Benzalkonium Chloride tolerance, and disinfectant resistance were not found in all strains, suggesting variability in environmental adaptability.

## 2 Materials and methods

### 2.1 Whole-genome sequencing and assembly

The raw sequence data of the strains in this study were obtained in a published study (Wei et al., 2024), from raw milk and pasteurized milk samples collected in three Ethiopian regions (i.e., Amhara, Oromia, and the Southern Nations, Nationalities, and Peoples’ Region (SNNPR)) between 2020 and 2021. Samples were collected from three stages of a dairy supply chain, including collectors, processors, and producers by Addis Ababa University. Samples were tested for *Listeria spp*., including *L. monocytogenes* by following the ISO 11290–1:2018 protocol and isolates were confirmed using a multiplex PCR (Chen and Knabel, 2007). The strains were sequenced by Michigan Department of Agriculture and Rural Development, submitted by Food and Drug Administration (FDA) GenomeTrakr Project and registered in the NCBI Sequence Read Archive (SRA) repository under the accession number PRJNA357724. For this study, the raw sequence data (which were saved as FASTQ file) for strains of 14 *L. monocytogenes* were retrieved from NCBI (BioProject: PRJNA357724), each strains was sequenced using the Illumina MiSeq instrument with v2 500 cycles kit (Illumina, San Diego, United States), producing paired-end reads. The quality of the raw sequence reads was assessed using fastQC v0.12.1. Sequencing data were processed to remove low-quality bases and adapter sequences with the optimized settings (LEADING:6 TRAILING:6 SLIDINGWINDOW:4:20 MINLEN:50) using Trimmomatic v0.39 (Bolger et al., 2014). After trimming, *de novo* assembly was performed using SPAdes v3.13.1 for downstream analysis such as identification of antimicrobial resistance and virulence genes. The quality of the assembly was determined using QUAST (Quality Assessment Tool for Genome Assemblies) and high-quality assemblies were included in the subsequent analyses.

Trimmed sequences were also aligned against the reference genome, *L. monocytogenes* EGD-e genome (Li and Durbin, 2009) with bwa v0.7.18 with the default options and filtered out by samtools v1.13. Variant calling was carried out using freebayes v0.9.12. Single Nucleotide Polymorphisms (SNPs) were extracted from the VCF file after applying stringent quality filters to ensure the reliability of variant calls. The filtering criteria included a minimum mapping quality (MAPQ) of ≥30, base quality (BQ) of ≥20, and read depth (DP) of ≥10. Variants with an allele frequency (AF) of ≥0.01 and a minimum alternative allele count (AC) of ≥2 were retained to minimize false positives. The filtered SNPs were then used to construct a phylogenetic tree to infer evolutionary relationships among the strains.

Multiple sequences alignment was performed using ClustalW. The phylogenetic tree was reconstructed using the Maximum Likelihood method based on the General Time Reversible (GTR) model (Nei and Kumar, 2000). For heuristic searches, initial tree topologies were obtained using the Neighbor-Join and BioNJ algorithms, applied to a matrix of pairwise distances estimated using the Maximum Composite Likelihood (MCL) approach, with the topology showing the highest log likelihood selected. Bootstrapping of 1,000 was used for confidence estimation. The analysis included 14 nucleotide sequences, and all positions with less than 95% site coverage were eliminated (partial deletion option), allowing for fewer than 5% alignment gaps, missing data, and ambiguous bases at any position. All evolutionary analyses were conducted using MEGA11 (Tamura et al., 2021).

### 2.2 Subtyping and core genome multilocus sequence typing of *Listeria monocytogenes*

MLST was conducted as recommended by the Institut Pasteur with seven housekeeping genes (*abcZ*, *bglA*, *cat*, *dapE*, *dat*, *ldh*, and *lhkA*). Serogroup PCR, Sequence Type (ST), clonal complex (CC) and Core Genome Multilocus Sequence Typing (cgMLST) were assigned from the *Listeria* MLST database (accessed on 26 August 2024 at https://bigsdb.pasteur.fr/listeria/) managed by the Institut Pasteur and based on Ragon’s scheme (Ragon et al., 2008; Moura et al., 2016). In addition, to ensure the robustness of the clustering results, we used MLST 2.0 (https://cge.food.dtu.dk/services/MLST/). A minimum spanning tree was constructed by using the software GrapeTree 1.5.0 (Zhou et al., 2018), and we have used the clustering parameters based on a seven allelic difference, as described by Moura et al. (2016), to define the genetic relationships among the isolates.

### 2.3 Detection of virulence, antimicrobial resistance, and stress, metal, and disinfectant resistance genes

Virulence genes in the strains of *Listeria monocytogesnes* were identified using the virulence factor database (VFDB) and National Center for Biotechnology Information (NCBI) *via* a local installation of Abricate v.1.0.1. Antimicrobial-resistant genes were also screened through the Comprehensive Antibiotic Resistance Database (CARD) (Alcock et al., 2023) and NCBI using a local installation of Abricate v.1.0.1. Abricate was configured with a minimum coverage of 80% and a minimum identity of 80%. Heatmaps were constructed using ggplot2 version 3.5.1 (R version 4.4.1) for both virulence and antimicrobial resistance genes. The change between resistant genes and reference genome was visualized using IGV (Integrative Genomics Viewer) v2.18.2.

Additionally, genes encoding for stress resistance, and metal and disinfectant resistance were detected using BLASTN algorithm according to BIGSdb-Lm database (Moura et al., 2016; Jolley and Maiden, 2010), available at https://bigsdb.pasteur.fr/listeria/ [accessed on 27 August 2024].

### 2.4 Detection of plasmids and mobile genetic elements

The presence of plasmids in *L. monocytogenes* strains was assessed using PlasmidFinder 2.1 (Camacho et al., 2009; Carattoli et al., 2014), accessed on 26 August 2024 at https://cge.food.dtu.dk/services/PlasmidFinder/, with criteria of 95% identity and 60% coverage. Additionally, MGEs were detected using the MobileElementFinder v1.0.3 tool (Johansson et al., 2020), accessed on 26 August 2024 at https://cge.food.dtu.dk/services/MobileElementFinder/, with a minimum identity and coverage of 95% and 60%, respectively.

### 2.5 CRISPR-Cas systems in *Listeria monocytogenes* genome

The search for and characterization of CRISPR arrays and their association with Cas proteins was determined with CRISPRCasFinder v4.3.2 (Couvin et al., 2018; Zhang et al., 2018), which is available at https://crisprcas.i2bc.paris-saclay.fr and accessed on 1 October 2024. The following default parameters were used: 23–55 bp repeated sequence length, 25–60 bp spacer length, 0.6–2.5 spacer sequence size as a function of repeated sequence size, a gap size between repeats of 25–60 bp, 20% nucleotide mismatch between repeats, 33% nucleotide mismatch for truncated Repeat, 100 bp size of flanking regions for each analyzed CRISPR array, and 60% maximum percentage similarity between spacers.

### 2.6 Metabolic pathway analysis of virulence genes

A KEGG (Kyoto Encyclopedia of Genes and Genomes) pathway analysis was performed to investigate the metabolic pathways and mechanisms associated with the virulence genes identified in *L. monocytogenes* strains. This analysis was conducted using DAVID (Database for Annotation, Visualization, and Integrated Discovery), accessible at https://david.ncifcrf.gov/summary.jsp [accessed on 13/10/2024]. Only virulence genes present in the KEGG database (*InlA* and *InlB*) were included in the metabolic pathway analysis.

## 3 Results

### 3.1 MLST analysis

All strains of *L. monocytogenes* were belonged to phylogenetic lineage I, sublineage SL2, and PCR-serogroup IVb. In addition, two different MLST sequence types were identified: ST2 (92.86%, n = 13 strains), ST45 (7.14%, n = 1). ST 2 was found across multiple regions, including Amhara (seven strains), Oromia (four strains), and SNNPR (four strains). It was predominantly associated with both processed food (seven strains) and raw food (6 strains). The majority of strains with ST 2 were collected at processors and collectors, with six strains found at each collection site. In contrast, ST 145 was found in only one strain, which came from Oromia, associated with raw food, and collected by a collector. Furthermore, In addition, all strains of *L. monocytogenes* were grouped into CC2 (Table 1) (Wei et al., 2024).

**TABLE 1:** Distribution of *Listeria monocytogenes* isolates in Region, food type and collection site of the food.

Isolates	Region	Food type	Collection site	ST	cgMLST complex type (CT)	Serogroup
SRR19688183/SAMN28661694	Amhara	Processed	Processer	2	CT8229	IVb
SRR19688184/SAMN28661693	Amhara	Processed	Processor	2	CT8229	IVb
SRR19739148/SAMN28661633	Oromia	Raw	Collector	2	CT14682	IVb
SRR19739149/SAMN28661632	Oromia	Raw	Collector	2	CT14682	IVb
SRR19739166/SAMN28661659	SNNPPR	Processed	Processer	2	CT8229	IVb
SRR19785111/SAMN28661702	Oromia	Raw	Producer	2	CT8229	IVb
SRR19785119/SAMN28661713	SNNPPR	Processed	Processer	2	CT8229	IVb
SRR19688187/SAMN28661688	Amhara	Raw	Collector	2	CT8229	IVb
SRR19739177/SAMN28661644	Oromia	Raw	Collector	145	CT375	IVb
SRR19785114/SAMN28661698	Amhara	Raw	Collector	2	CT8229	IVb
SRR19785124/SAMN28661689	Amhara	Raw	Collector	2	CT8229	IVb
SRR19688182/SAMN28661695	Amhara	Processed	Processer	2	CT8229	IVb
SRR19739164/SAMN28661661	SNNPPR	Processed	Processer	2	CT8229	IVb
SRR19739165/SAMN28661660	SNN	Processed	Processer	2	CT8229	IVb

### 3.2 cgMLST analysis

**FIGURE 1:**
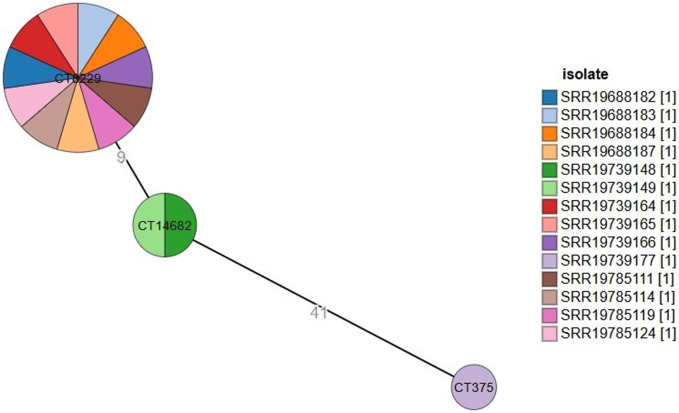
Minimum spanning tree constructed based on MLST allelic profiles of 14 *Listeria monocytogenes* isolates. Each circle clusters a cgMLST complex type, and colors distinguish isolates. Branch length indicates the difference in the number of alleles.

Based on the cgMLST analysis, *L. monocytogenes* strains were classified into three different CTs (Figure 1). The most prevalent one, cgMLST type CT8229, covered 11 strains obtained from three different regions (Amhara, Oromia and SNNPR), both raw and pasteurized milk, and three different milk sources (collector, processor and producer). The remaining cgMLST types were CT14682 (found in two strains), and CT375 (found in one strains), both of which were obtained only from Oromia region, raw milk, and milk collectors (Table 1).

The minimum spanning tree (Figure 1) showed how various strains relate to one another in terms of genetic distance, using clustering parameters based on a seven allelic difference, as described by Moura et al. (2016). CT9229 forms a large and diverse cluster, suggesting that the strains within this cluster are genetically similar but show some variability. It contains many strains that are closely related, possibly indicating a recent common ancestor or a rapidly evolving population. CT14682 is smaller and contains fewer strains. The genetic distance of nine between CT9229 and CT14682 suggests that these two clusters are relatively close but distinct, possibly indicating separate evolutionary events or selective pressures. CT375 is isolated and has a large genetic distance of 41 from CT14682, indicating that this strain has evolved more independently.

### 3.3 Virulence genes in the studied *Listeria monocytogenes* strains

**FIGURE 2:**
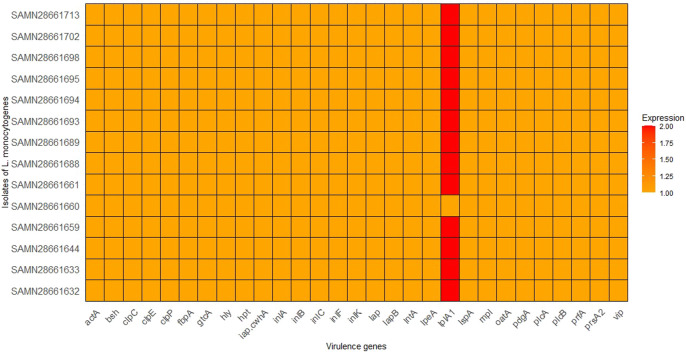
Distribution of virulence genes in the 14 isolates of *L. monocytognes*. Isolates are represented on the y-axis, the x-axis represents the virulence genes being analyzed, and the color intensity corresponds to the number of copies of each virulence gene found in each isolate.

To investigate the virulence potentials of the 14 strains, an *in silico* detection of 29 virulence genes was carried out, main virulent genes in each strain were presented in Figure 2. All virulence genes were detected in all the 14 *L. monocytogenes* strains. However, *lplA1* is detected in higher amount in all strains, except SAMN28661660, suggesting it may play a significant role in the virulence of these particular strains. This suggests that lplA1 is the most distinctively expressed virulence gene among the strains. Thus, the lplA1 could be the most effective genetic sequence for continuous environmental monitoring. This gene could serve as a valuable marker for rapid testing and surveillance, potentially aiding in early outbreak detection.

### 3.4 Antibiotic resistance genes in the studied *Listeria monocytogenes* strains

**FIGURE 3:**
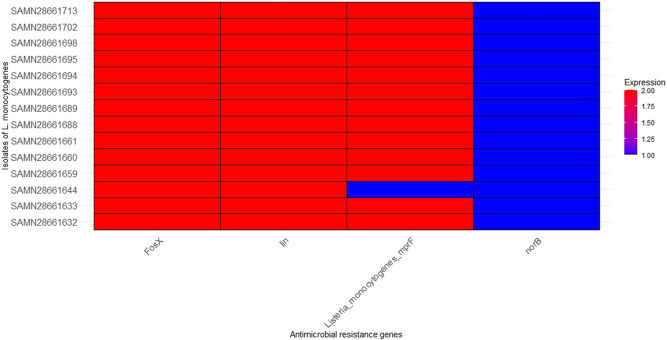
Distribution of antimicrobial resistance genes in the 14 isolates of *L. monocytognes*. Isolates are represented on the y-axis. The x-axis represents specific antimicrobial resistance genes involved in resistance mechanisms. The color intensity corresponds to the number of copies of each antimicrobial resistance genes found in each isolate.

Four antibiotic resistance genes were identified in this study (Figure 3). *fosX* (resistance to fosfomycin), *lin* (resistance to lincosamides), *norB* (resistance to quinolones), and *Listeria_monocytogenes_mprF* Cationic peptide (resistance to cationic antimicrobial peptides) were present in all studied strains of *L. monocytogenes*. *fosX*, *Listeria_monocytogenes_mprF*, and *lin* were detected in high level in all strains, except low level detection of *lin* in SAMN28661644. This suggests that these resistance genes are highly conserved and potentially critical for the resistance profile of these *L. monocytogenes* strains. *norB* was identified in a lower amount in all strains.

### 3.5 Genes for stress, metal, and disinfectant resistance and genomic island

In this study, all 14 *L. monocytogenes* strains were detected for the presence of stress, metal, and disinfectant resistance adaptation associated genes or gene clusters. Stress resistance genes include *stress survival islet 1* (*SSI1_lmo0447*), *lmo 1800*, and *lmo1799* were identified in all strains of *L. monocytogenes*, whereas, none of the strains harboured *Stress Survival Islet 2* (*STI2*). However, genes encoding for metal/Benzalkonium Chloride tolerance and disinfectant resistance were not identified from all 14 strains of *L. monocytogenes*. LGI-2 was found in all the strains and none of the studied strains harbored LGI-1 and LGI-3.

### 3.6 Plasmids and mobile genetic elements

The presence of plasmids in *L. monocytogenes* strains was tested using PlasmidFinder 2.1. However, plasmids were not detected in any of the 14 strains. Additionally, MGEs were assessed using MGEs, but none were found in any of the 14 strains.

### 3.7 Phylogenetic relationship of strains

**FIGURE 4:**
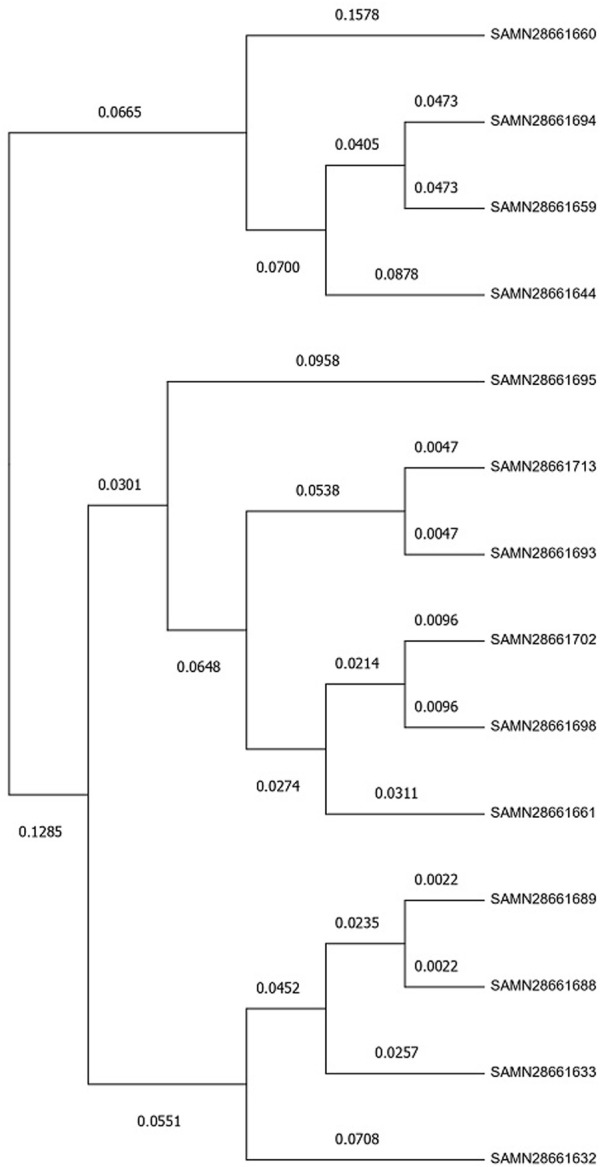
Evolutionary analysis by Maximum Likelihood method. The evolutionary history was inferred by using the Maximum Likelihood method and General Time Reversible model (Nei and Kumar, 2000). The tree with the highest log likelihood (−8,483.46) is shown. The percentage of trees in which the associated taxa clustered together is shown next to the branches. Initial tree(s) for the heuristic search were obtained automatically by applying Neighbor-Join and BioNJ algorithms to a matrix of pairwise distances estimated using the Maximum Composite Likelihood (MCL) approach, and then selecting the topology with superior log likelihood value. A discrete Gamma distribution was used to model evolutionary rate differences among sites (five categories (+G, parameter = 9.4062)). The rate variation model allowed for some sites to be evolutionarily invariable ([+I], 0.00% sites). This analysis involved 14 nucleotide sequences. All positions with less than 95% site coverage were eliminated, i.e., fewer than 5% alignment gaps, missing data, and ambiguous bases were allowed at any position (partial deletion option). There were a total of 1,513 positions in the final dataset. Evolutionary analyses were conducted in MEGA11 (Tamura et al., 2021).

SNP based phylogenetic tree (Figure 4) was constructed using MEGA 11 that represented the evolutionary relationships among the 14 strains of *L. monocytogenes* based on their genetic similarity. Closely related samples like SAMN28661688 and SAMN28661689 (with a genetic distance of 0.0022) likely share a recent common ancestor, whereas SAMN28661660 showed greater divergence, indicating it may have evolved separately from the other samples over a longer period.

### 3.8 CRISPR-Cas systems

Analysis of the CRISPR-Cas system of strains revealed that most strains have two CRISPR arrays located at similar genome positions, with consistent repeat consensus sequences: “TTTCCACCACCACCTACGGATGAAGAGTTAAGACTTGCTTTGCCAGAGAC” and “GAACTCGTATCAAAACTGCTTAA.” 14.28% (2/14) of the strains also carry the Cas gene csa5, indicating a functional Cas system (CAS-TypeIA) (Table 2). The presence of csa5 in a subset of strains indicates potential variability in CRISPR-Cas activity, with some strains possibly retaining an active defense mechanism against foreign genetic elements, such as phages and plasmids, while others may rely on alternative defense strategies. The overall conservation of CRISPR arrays and their structural features suggests that this system may play a significant role in genome stability and strain adaptation, potentially influencing their evolutionary trajectory and resistance to horizontal gene transfer.

**TABLE 2:** CRISPR-Cas systems identified in *Listeria monocytogenes* genomes.

Isolates	OST	Position	Max. spacer/strain	Arr/strain	Repeat consensus	Cas genes
SAMN28661688	CRISPR Array	123268-123422	1	1	TTTCCACCACCACCTACGGATGAAGAGTTAAGACTTGCTTTGCCAGAGAC	
		46679-46857	2	1	GAACTCGTATCAAAACTGCTTAA	
SAMN28661693	CRISPR Array	46679-46857	2	1	GAACTCGTATCAAAACTGCTTAA	
		123268-123422	1	1	TTTCCACCACCACCTACGGATGAAGAGTTAAGACTTGCTTTGCCAGAGAC	
SAMN28661695	CRISPR Array	46679-46857	2	1	GAACTCGTATCAAAACTGCTTAA	
		123268-123422	1	1	TTTCCACCACCACCTACGGATGAAGAGTTAAGACTTGCTTTGCCAGAGAC	
SAMN28661694	CRISPR Array	46679-46857	2	1	GAACTCGTATCAAAACTGCTTAA	
		123268-123422	1	1	TTTCCACCACCACCTACGGATGAAGAGTTAAGACTTGCTTTGCCAGAGAC	
SAMN28661661	CRISPR Array	46679-46857	2	1	GAACTCGTATCAAAACTGCTTAA	
		123268-123422	1	1	TTTCCACCACCACCTACGGATGAAGAGTTAAGACTTGCTTTGCCAGAGAC	
SAMN28661644	CRISPR Array	478275-478429	1	1	TTTCCACCACCACCTACGGATGAAGAGTTAAGACTTGCTTTGCCAGAGAC	
		46678-46856	2	1	GAACTCGTATCAAAACTGCTTAA	
SAMN28661633	CAS-TypeIA	122603-122757	1	1	TTTCCACCACCACCTACGGATGAAGAGTTAAGACTTGCTTTGCCAGAGAC	csa5
		46679-46857	2	1	GAACTCGTATCAAAACTGCTTAA	
SAMN28661660	CRISPR Array	46679-46857	2	1	GAACTCGTATCAAAACTGCTTAA	
		123268-123422	1	1	TTTCCACCACCACCTACGGATGAAGAGTTAAGACTTGCTTTGCCAGAGAC	
SAMN28661659	CRISPR Array	46679-46857	2	1	GAACTCGTATCAAAACTGCTTAA	
		123268-123422	1	1	TTTCCACCACCACCTACGGATGAAGAGTTAAGACTTGCTTTGCCAGAGAC	
SAMN28661632	CAS-TypeIA	46679-46857	2	1	GAACTCGTATCAAAACTGCTTAA	csa5
		123269-123423	1	1	TTTCCACCACCACCTACGGATGAAGAGTTAAGACTTGCTTTGCCAGAGAC	
SAMN28661689	CRISPR Array	46679-46857	2	1	GAACTCGTATCAAAACTGCTTAA	
		123268-123422	1	1	TTTCCACCACCACCTACGGATGAAGAGTTAAGACTTGCTTTGCCAGAGAC	
SAMN28661702	CRISPR Array	46679-46857	2	1	GAACTCGTATCAAAACTGCTTAA	
		123268-123422	1	1	TTTCCACCACCACCTACGGATGAAGAGTTAAGACTTGCTTTGCCAGAGAC	
SAMN28661698	CRISPR Array	46679-46857	2	1	GAACTCGTATCAAAACTGCTTAA	
		123268-123422	1	1	TTTCCACCACCACCTACGGATGAAGAGTTAAGACTTGCTTTGCCAGAGAC	
SAMN28661713	CRISPR Array	46679-46857	2	1	GAACTCGTATCAAAACTGCTTAA	
		123268-123422	1	1	TTTCCACCACCACCTACGGATGAAGAGTTAAGACTTGCTTTGCCAGAGAC	

**Key**: OST, open structure type; Max. Spacer/Strain, Maximum number of Spacers per strain; Arr/Strain, Array per strain.

### 3.9 Metabolic pathway analysis

**FIGURE 5:**
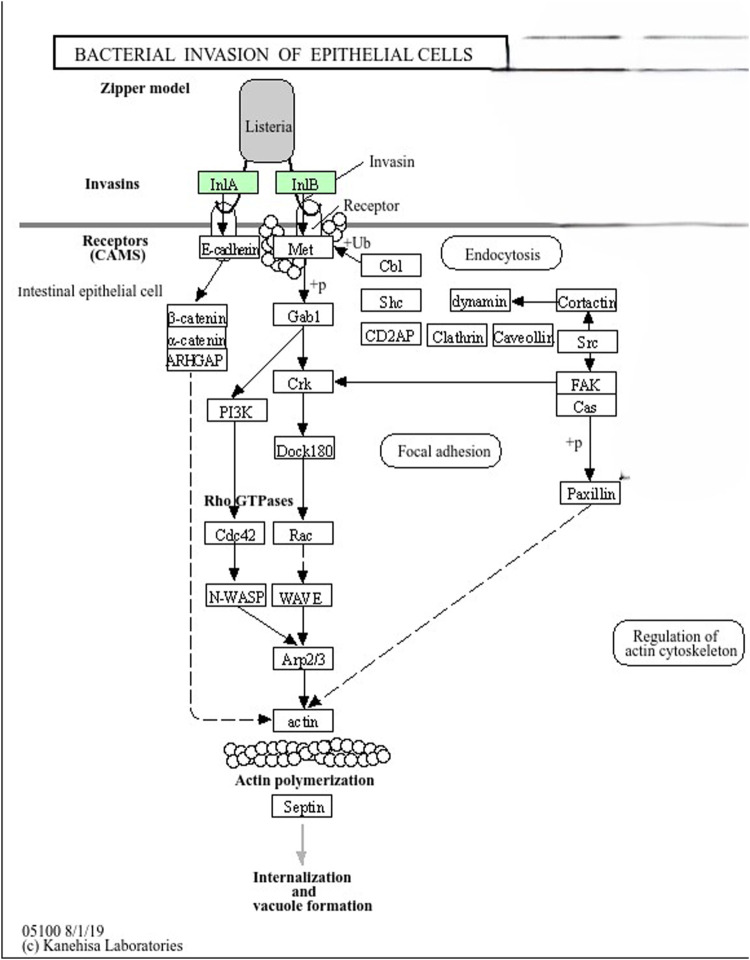
*InlA* and *InlB* in Zipper Model (*Listeria* Invasion Pathway). *Listeria monocytogenes* uses *InlA* and *InlB* (colored in green) proteins as invasins to bind receptors like E-cadherin and Met on the surface of intestinal epithelial cells. This interaction triggers actin polymerization through a cascade of signaling molecules, including PI3K, Rho GTPases (Cdc42, Rac), N-WASP, and the Arp2/3 complex, leading to internalization of the bacterium. The actin cytoskeleton is regulated to facilitate bacterial entry and vacuole formation.

A KEGG pathway analysis was performed to investigate the metabolic pathways and mechanisms associated with the virulence genes identified in *L. monocytogenes* strains using DAVID. *InlA* and *InlB* are used by *L. monocytogenes* as invasins to bind receptors like E-cadherin and Met on the surface of intestinal epithelial cells. This interaction triggers actin polymerization through a cascade of signaling molecules, including PI3K, Rho GTPases (Cdc42, Rac), N-WASP, and the Arp2/3 complex, leading to internalization of the bacteria. The actin cytoskeleton is regulated to facilitate bacterial entry and vacuole formation (Figure 5). The enrichment of *L. monocytogenes* virulence genes in the actin polymerization pathway indicates that actin dynamics are essential for the bacteria’s ability to enter host cells and cause infection. Understanding how the bacteria manipulate this pathway can lead to the development of more accurate diagnostic tools, such as biomarkers specific to this pathway, enabling sensitive detection of *L. monocytogenes* infections. Additionally, targeting key components of the actin polymerization pathway could provide new therapeutic strategies to prevent bacterial entry and control infection.

## 4 Discussion

In Ethiopia, *L. monocytogenes* contamination in milk and dairy products is alarmingly high, with some studies reporting rates up to 60% (Tola, 2024; Seyoum et al., 2015). This poses a significant public health risk, given the widespread consumption of dairy, particularly raw milk, in both rural and urban areas as well as inadequate sanitation in dairy processing and poor handling practices (Abdi Keba et al., 2020; Mebrate et al., 2020). Its ubiquity, persistence under adverse environmental conditions, pathogenicity (Hurley et al., 2019; Chen et al., 2020), and ability to develop multidrug resistance (Dufailu et al., 2021) contribute to its impact. Understanding the pathogen’s virulence, antimicrobial resistance mechanisms, and environmental stress adaptation in the region/Ethiopia is crucial for developing novel, efficient, and cost-effective antimicrobial agents and biocontrol methods. In this study, we applied WGS to strains isolated from milk and dairy products in Ethiopia to examine their virulence gene profiles, antimicrobial resistance genes, MGEs linked to virulence and resistance, and genetic relatedness.

A total of 14 *L. monocytogenes* strains originating from milk and dairy products were typed and characterized for the presence of virulence, antimicrobial resistance, and stress response genetic determinants. MLST typing in this study revealed a dominance of sequence type ST2 (92.86%, n = 13) over ST45 (7.14%, n = 1), aligning with previous research suggesting that certain clones, such as ST2, are more prevalent in food-related environments, including dairy products (Amarasekara et al., 2024). The widespread occurrence of ST2 in food sources is further supported by Tricoli et al. (2024), who reported the circulation of ST2, a clinically relevant strain linked to suspected food contamination.

All strains belonged to PCR-serogroup IVb and were classified into clonal complex (CC) 2, further supporting the notion that ST2 strains are well adapted to persist in food production settings (Koopmans et al., 2023). CC2, identified in the studied strains, is one of the dominant clonal complexes responsible for listeriosis in different regions of the world (Chenal-Francisque et al., 2011). This complex is known to confer hypervirulence by enhancing bacterial invasion into the central nervous system (CNS) and the placenta, which significantly increases the risk of severe infections (Maury et al., 2016).

While ST45 was less common in this study, it has been reported in China as spreading across different regions, demonstrating remarkable virulence potential. Specifically, ST45 strains exhibited high hemolysin activity and significant virulence capacity, particularly in bloodstream infections (Wang et al., 2019). These findings emphasize the importance of continuous monitoring and characterization of *L. monocytogenes* strains in dairy products to better understand their potential risks to public health.

The cgMLST analysis revealed three distinct core genome sequence types (CTs) with the most prevalent type, L1-SL2-ST2-CT8229, comprising 11 strains from diverse geographical regions (Amhara, Oromia, South Nation Nationalities), different types of milk (raw and pasteurized), and various sources within the dairy supply chain (collectors, processors, and producers). This widespread distribution of CT8229 suggests a highly adaptive clone that may be circulating widely in Ethiopia’s milk production chain. The broad presence of this cgMLST type across multiple regions and milk sources highlights the potential for cross-contamination at different points in the supply chain, indicating gaps in food safety practices that require attention (Palaiodimou et al., 2021). The less prevalent cgMLST types, L1-SL2-ST2-CT14682 and L1-SL2-ST45-CT375, were found only in raw milk from the Oromia region, specifically from milk collectors. This suggests a more localized and specific distribution pattern, possibly linked to regional practices in milk collection and handling (Maury et al., 2016).

The minimum spanning tree (MST) analysis (Figure 1) shows that CT8229 forms a large and diverse cluster, indicating genetic homogeneity with slight variability. This genetic closeness may suggest a recent common ancestor or rapid clonal expansion in response to selective pressures within the dairy production environment. Such expansion may be due to environmental resilience, increased fitness, or adaptation to selective pressures like pasteurization or other food processing measures (Henri et al., 2016). The genetic distance of nine between CT8229 and CT14682 indicates that these clusters are genetically close, possibly reflecting micro-evolutionary changes or selective pressures that have shaped their genomic characteristics. CT14682 may represent a slightly divergent subgroup of ST2, indicating the evolution of sub-clonal populations that could respond differently to environmental pressures or interventions. In contrast, the genetic distance of 41 between CT14682 and CT375, which belongs to ST45, suggests that this strain has evolved more independently. The significant divergence of CT375 may reflect distinct evolutionary events, potentially driven by different ecological niches or selective pressures in the dairy environment (Osek et al., 2022).

The detection of all 29 virulent and virulence-related genes across all 14 *L. monocytogenes* strains underscores the virulent potential of these strains. This result is consistent with previous studies where the core virulence genes of *L. monocytogenes*, such as *prfA*, *hly*, *actA*, and *inlA*, were present in all strains regardless of their source (Maury et al., 2016). A particularly interesting finding is the elevated detection of *lplA1* in all strains except SAMN28661660. *lplA1* is associated with lipid metabolism and has been implicated in the virulence of *L. monocytogenes* during host infection, contributing to intracellular survival and growth (Harter et al., 2019). Its high prevalence in the strains may indicate a critical role in the virulence of these strains, potentially aiding in the persistence and pathogenicity of *L. monocytogenes* in different environments. This also suggests that *lplA1* is the most distinctively expressed virulence gene among the strains. Thus, the *lplA1* could be the most effective genetic sequence for continuous environmental monitoring. This gene could serve as a valuable marker for rapid testing and surveillance, potentially aiding in early outbreak detection. The absence of this gene in SAMN28661660 may indicate differences in its virulence potential, warranting further investigation into how *lplA1* influences the overall virulence and fitness of *L. monocytogenes* in various ecological niches.

Four antibiotic resistance genes—*fosX*, *lin*, *norB*, and *mprF*—were identified across all strains. The consistent presence of *fosX* (fosfomycin resistance), *mprF* (cationic antimicrobial peptide resistance), and *lin* (lincosamide resistance) suggests these genes are highly conserved in *L. monocytogenes* populations, potentially contributing to the persistence of antibiotic resistance in the environment (Morvan et al., 2010). The lower copy number detection of *lin* in SAMN28661644 could reflect a varying degree of resistance expression or a genetic variation specific to this strain. Meanwhile, *norB* (quinolone resistance) was detected at a lower copy number across all strains, indicating it may not be as critical for resistance as the other genes, but its presence still poses a concern for treatment efficacy, especially in clinical settings where quinolones are used (Parra-Flores et al., 2022). These findings reflect the importance of continuous monitoring of antimicrobial resistance genes in *L. monocytogenes* to mitigate the risk of resistance spread. In agreement to this study, Parra-Flores et al. (2022) reported the presence of resistance genes with mechanisms of antibiotic efflux (norB), antibiotic target alteration (mprF), and antibiotic inactivation (lin, fosX). However, in addition, their study reported genes that confer resistance to tetracycline (tetA and tetC).

All strains harbored *stress survival islet 1* (*SSI1_lmo0447*) and the *lmo1799* and *lmo1800* genes, which are known to confer stress resistance, particularly in low pH or osmotic stress conditions that are common in food processing environments (Bergholz et al., 2012). This widespread presence indicates that these strains are well-equipped to survive harsh conditions, which is a concern for food safety as *L. monocytogenes* can persist even in processed food products. However, the absence of *stress survival islet 2* (*SSI2*) in all strains is noteworthy since *SSI2* is linked to survival under alkaline and oxidative stress conditions. Its absence may suggest that the strains in this study are less adapted to these specific stresses, possibly due to the particular conditions of the milk production environment from which they were isolated (Harter et al., 2017).

The absence of genes encoding resistance to metals and disinfectants, including benzalkonium chloride, in all strains is a positive finding. Benzalkonium chloride is commonly used in food production as a disinfectant, and its effectiveness against *L. monocytogenes* in strains of this study suggests that these strains may not have developed resistance mechanisms that could undermine food safety efforts (Romanova et al., 2006). This finding emphasizes the continued relevance of current disinfection practices in dairy production facilities for controlling *L. monocytogenes*.

All strains carried the *Listeria* genomic island 2 (LGI-2), while none harbored LGI-1 or LGI-3. LGI-2 is known to contribute to heavy metal resistance, antibiotic resistance, and stress adaptation (Kuenne et al., 2010), which could explain the persistence of these strains in the dairy environment. Despite the presence of LGI-2 in these strains, the absence of predicted heavy metal resistance genes indicates that these particular strains have not yet developed mechanisms to overcome this specific disinfectant. This highlights a critical aspect of microbial evolution, where resistance to certain agents may develop independently and is influenced by selective pressures (Christaki et al., 2020). However, the absence of plasmids, as revealed by PlasmidFinder, suggests that plasmid-mediated transfer of resistance or virulence factors is not occurring in this population. Similarly, no MGEs were identified, which is an encouraging sign as MGEs, including transposons and integrative conjugative elements, play a key role in the horizontal transfer of resistance genes (Pricope et al., 2013). This might indicate that the genetic content of the strains is relatively stable, with no immediate threat of gene transfer to other bacterial populations.

The SNP-based phylogenetic tree constructed using MEGA 11 illustrates the evolutionary relationships between the 14 *L. monocytogenes* strains. The close genetic distance between samples SAMN28661688 and SAMN28661689 suggests a recent common ancestor, which may imply shared evolutionary pressures or sources of contamination. In contrast, SAMN28661660 shows greater genetic divergence, suggesting it evolved separately from the other strains over a longer period. Phylogenetic trees based on SNP data have been widely used to infer the evolutionary relationships and trace the transmission patterns of *L. monocytogenes* in both clinical and environmental settings (Chen et al., 2020). This tree further underscores the genetic heterogeneity present in the studied strains, which can have implications for their virulence, resistance, and ability to survive in diverse environments.

CRISPR-Cas systems are acquired immunity systems that allow bacteria and archaea to acquire exogenous material from bacteriophages and plasmids (Hupfeld et al., 2018). The CRISPRCas systems is a possible involved in the regulation of gene expression, including virulence genes, which have been described in a number of pathogens (Louwen et al., 2014). In this study, the analysis of the CRISPR-Cas system revealed that most strains harbor two CRISPR arrays with conserved repeat sequences, indicating a relatively stable CRISPR system in these strains. CRISPR systems, particularly type IA, play important roles in bacterial immunity by providing resistance to foreign genetic elements, such as bacteriophages or plasmids (Marraffini, 2013). The presence of the Cas gene *csa5* in 14.28% of the strains suggests a functional CRISPR-Cas system, which could enhance the adaptability of these strains by defending against horizontal gene transfer. This functionality may contribute to the evolutionary success of *L. monocytogenes*, as it can protect against phage attacks or acquisition of harmful MGEs while maintaining virulence potential. CRISPR-Cas system plays multiple roles beyond adaptive immunity, i.e., gene regulation and virulence (Faure et al., 2019). Understanding the structure and function of CRISPRCas system contributes to develop useful technology and products to control pathogen, i.e., phages of *Listeria* offer novel tools for detection, differentiation, CRISPR-Cas-assisted phage engineering, diagnostics, and biocontrol (Hagens and Loessner, 2014; Hupfeld et al., 2018; Meile et al., 2020).

The KEGG pathway analysis highlights the molecular mechanisms through which *L. monocytogenes* invades host cells. The virulence genes *inlA* and *inlB* were shown to play central roles in ability of the pathogen to bind to host cell receptors like E-cadherin and Met. This interaction activates a signaling cascade involving PI3K, Rho GTPases, N-WASP, and the Arp2/3 complex, which promotes actin polymerization and leads to bacterial internalization into host cells (Pizarro-Cerdá et al., 2012). The manipulation of the host actin cytoskeleton is a well-established strategy used by *L. monocytogenes* to evade immune responses and spread within the host, making it a key factor in the pathogen’s ability to cause infection. The conserved nature of these virulence mechanisms across the strains suggests that targeting these pathways could be a potential avenue for therapeutic intervention in *L. monocytogenes* infections. On the other hand, research indicates that while intact *inlA* genes are prevalent in clinical strains, truncations often occur in isolates from food processing environments, primarily due to premature stop codons (PMSCs) (Gorski et al., 2016). In food isolates, five out of seven cultures exhibited truncated *inlA* profiles, contrasting with the intact profiles found in clinical isolates (Tamburro et al., 2015).

## 5 Limitation of the study

The key limitations of this study is the relatively small sample size of *L. monocytogenes* strains analyzed, which includes only 14 strains from milk and dairy products in Ethiopia. This limited number of strains may not fully represent the genetic diversity of *L. monocytogenes* in different regions, production methods, or types of dairy products across the country. As a result, the findings may not be entirely generalizable to the broader population of *L. monocytogenes* in Ethiopia or other regions with similar agricultural and dairy production environments. Future studies should incorporate more diverse geographic and environmental samples to strengthen conclusions. Such an approach would provide a more comprehensive understanding of *L. monocytogenes* diversity, antimicrobial resistance patterns, and potential public health risks.

Despite our genomic findings provide valuable insights regarding antibiotic resistance genes, there was no experimental validation (e.g., antimicrobial susceptibility testing) to confirm resistance phenotypes. Therefore, future studies incorporating phenotypic validation will be necessary to confirm the functional resistance conferred by these genes. In addition, although this study indicates the presence of CRIPR-Cas system, experimental verification of CRISPR activity there was not performed. As a result, future work using functional assays, such as phage challenge experiments or plasmid interference assays, would be necessary to confirm the protective role of CRISPR-Cas in *L. monocytogenes*.

## 6 Conclusion and recommendations

This study highlights the significant genomic diversity among *L. monocytogenes* strains from dairy products in Ethiopia. The strains were categorized into two sequence types (ST2 and ST45), with ST2 being the dominant type. All 14 strains examined contained a complete set of 29 virulence genes, suggesting they may have a potential for pathogenicity. Additionally, antibiotic resistance genes associated with fosfomycin, lincosamides, and quinolones were identified, suggests a potential for antibiotic resistance in *L. monocytogenes* within the dairy sector. All strains harbored LGI-2, while none contained LGI-1 or LGI-3, nor did they possess genes related to metal resistance, disinfectant tolerance, or MGEs. The presence of the CRISPR-Cas system in some strains may contribute to phage defense and the preservation of genetic integrity. Additionally, KEGG pathway analysis illuminated the molecular mechanisms underlying host cell invasion, emphasizing the pathogen’s virulence potential. While this study provides important insights, it was conducted using a relatively small sample size of only 14 strains. Future research should expand the scope by including a larger number of isolates to better understand the full diversity of *L. monocytogenes* in dairy products. Further studies should investigate the role of CRISPR-Cas systems and other genetic elements in bacterial virulence and antimicrobial resistance. Moreover, continuous monitoring of *L. monocytogenes* in the dairy sector, coupled with stricter sanitation practices, food safety regulations, and targeted public health interventions, is crucial to mitigate the risks of contamination and resistance spread.

## Data Availability

The datasets used and/or analyzed during the current study are available in the NCBI Sequence Read Archive (SRA) repository under the accession number PRJNA357724.

## Abbreviations

BLASTN, Basic Local Alignment Search Tool for Nucleotide Sequences; CARD, Comprehensive Antibiotic Resistance Database; CC, clonal complex; CgMLST, Core Genome Multilocus Sequence Typing; CRISPR-Cas, Clustered Regularly Interspaced Short Palindromic Repeats-CRISPR-associated proteins; DAVID, Database for Annotation, Visualization, and Integrated Discovery; EGD, *Epidemic Group D*; FASTQ, Fast Quality Format; IGV, Integrative Genomics Viewer; KEGG, Kyoto Encyclopedia of Genes and Genomes; *L. monocytogenes*, *Listeria* monocytogenes; LGI-1, *Listeria* Genomic Island 1; LGI-2, *Listeria* Genomic Island 2; LGI-3, *Listeria* Genomic Island 3; MGEs, Mobile Genetic Elements; MLST, Multilocus Sequence Typing; NCBI, National Center for Biotechnology Information; QUAST, Quality Assessment Tool for Genome Assemblies; SNNPR, Southern Nations, Nationalities, and Peoples’ Region; SNP, Single-nucleotide polymorphism; SRA, Sequence Read Archive; ST, Sequence Type; VCF, Variant Call Format; VFDB, virulence factor database; WGS, whole-genome sequencing.
